# ﻿Diversity of fungi associated with *Monochamusalternatus* larval habitats in *Bursaphelenchusxylophilus*-infected *Pinusmassoniana* and identification of two new ophiostomatalean species (Ascomycota, Ophiostomatales)

**DOI:** 10.3897/mycokeys.92.80682

**Published:** 2022-08-01

**Authors:** Guiheng Zheng, Minqi You, Xuening Li, Qinzheng Zhou, Zheng Wang, Huimin Wang, Quan Lu

**Affiliations:** 1 Key Laboratory of Forest Protection, National Forestry and Grassland Administration; Ecology and Nature Conservation Institute, Chinese Academy of Forestry, Beijing 100091, China Ecology and Nature Conservation Institute, Chinese Academy of Forestry Beijing China; 2 Agriculture and Rural Affairs Bureau of Huangyan District, Taizhou City 318020, China Agriculture and Rural Affairs Bureau of Huangyan District Zhejiang China; 3 Research Institute of Desertification, Chinese Academy of Forestry, Beijing 100091, China Research Institute of Desertification, Chinese Academy of Forestry Beijing China

**Keywords:** *Ceratocystiopsis*, fungal succession, *Graphilbum*, *Ophiostoma*, pine wilt disease, *Sporothrix*, two new species

## Abstract

*Bursaphelenchusxylophilus*, a pathogenic pine wood nematode (PWN), is responsible for pine wilt disease (PWD), which has caused significant economic and ecological damage worldwide, particularly in East Asia. Multiple biological factors, such as the beetle vector *Monochamus*, symbiotic bacteria and associated fungi, are involved in the disease infection cycle. This study isolated and identified the fungal communities of *Monochamusalternatus* larval galleries and pupal chambers from different instars through field investigation, morphological observation and multi-locus DNA sequence analyses in Zhejiang Province, China. A total of 255 and 454 fungal strains were isolated from *M.alternatus* galleries and pupal chambers infected with PWN, from the 2^nd^–3^rd^ and 4^th^–5^th^ instar larvae, respectively. A total of 18 species of fungi were identified, 14 species were isolated from the 2^nd^–3^rd^ instar larval galleries and six species from the galleries and pupal chambers of the 4^th^–5^th^ instar larvae. Amongst them were six species belonging to four genera of ophiostomatalean fungi, including two novel species, *Graphilbumxianjuensis***sp. nov.** and *Ophiostomataizhouense***sp. nov.** and four known species, *Ceratocystiopsisweihaiensis*, *Ophiostomaips*, *Sporothrixzhejiangensis* and *S.macroconidia*. The findings revealed that the fungal diversity and abundance of the 2^nd^–3^rd^ instar larvae differed markedly from those of the 4^th^–5^th^ instar larvae. This difference could be the result of fungal succession. This study provides a thorough understanding of the fungi associated with PWD and lays the groundwork for future research.

## ﻿Introduction

The pine wood nematode (PWN), *Bursaphelenchusxylophilus* (Steiner & Buhrer) Nickle, is a pathogenic nematode that is responsible for the devastating epidemic of pine wilt disease (PWD) worldwide (Mota and Vieira 2008; Robertson et al. 2011), particularly throughout Japan, Korea and China (Togashi and Jikumaru 2007; Jung et al. 2010; Abelleira et al. 2011; Foit et al. 2019). Since the first report in Nanjing, China, in 1982, PWD has spread through more than 700 counties in 19 provinces (State Forestry Administration of the People’s Republic of China 2021), killing over one billion pine trees (Zhu et al. 2021). Economic and ecological losses have totalled thousands of billions of Chinese Yuan. PWN has diverse carriers and hosts. Carriers include more than eight beetle species and at least 25 hosts are susceptible under natural conditions (Zheng et al. 2021). In China, the primary PWN vector is the sawyer beetle *Monochamusalternatus* Hope (Coleoptera, Cerambycidae), while *Pinusmassoniana* is one of the earliest and most susceptible hosts (Linit et al. 1983; Kobayashi et al. 1984; Ye 2019; Ji et al. 2021).

The PWN is the predominant pathogen in this complex ecosystem (Zhao et al. 2014). The symbiotic interaction between the PWN-vector and fungi is a key biological factor that promotes PWN pathogenicity and invasiveness (Zhao et al. 2014; Zhao and Sun 2017). Molecular analysis has repeatedly demonstrated that the fungus and the PWN have a close symbiotic relationship, as evidenced by the draft genome sequence of a PWN inbred line which revealed that all PWN cellulases were most likely acquired independently from fungi (Kikuchi et al. 2011). Metagenomic analysis of the PWN microbiome indicates that the PWN and its microbiome have established a potentially mutualistic symbiotic relationship with complementary pathways in detoxification metabolism (Kikuchi et al. 2011; Cheng et al. 2013).

Current research has shown that PWN has an important mycetophagous phase in its life history (Ryss et al. 2005; Espada et al. 2016). Many airborne fungi, including endophytes (*Botrytiscinerea* and *Cladosporiumherbarum*) and pathogens (*Sirococcusconigenus* and *Sphaeropsissapinea*), are positively correlated with the growth of the nematode population (Pimentel et al. 2021), with ophiostomatalean fungi (Ascomycota: Sordariomycetes: Ophiostomatales) particularly important in terms of their association with PWN-*M.alternatus* symbionts. The Ophiostomatales order includes one family (Ophiostomataceae) and twenty genera (*Afroraffaelea*, *Aureovirgo*, *Ceratocystiopsis*, *Chrysosphaeria*, *Dryadomyces*, *Esteya*, *Fragosphaeria*, *Graphilbum*, *Grosmannia*, *Hawksworthiomyces*, *Harringtonia*, *Heinzbutinia*, *Intubia*, *Jamesreidia*, *Leptographium*, *Masuyamyces*, *Ophiostoma*, *Paleoambrosia*, *Raffaelea* and *Sporothrix*) (de Beer et al. 2014, 2016, 2022; Hyde et al. 2020; Wijayawardene et al. 2022). *Sporothrix* sp.1, for example, induces the xylem tissue of the pine tree to produce diacetone alcohol, which may increase PWN propagation and beetle larvae growth (Zhao et al. 2013). PWN produces ascarosides that promote fungal (*Leptographiumpini-densiflora* and *Sporothrix* sp.1) growth and sporulation (Zhao et al. 2018). In addition, some fungi are detrimental to PWN. *Esteyavermicola* is an example of direct antagonism (Wang et al. 2011). The lunate conidia of *E.vermicola* are highly infectious to PWN (Liou et al. 1999).

The invasion of beetles altered the internal habitat and mycoflora of pine trees (Zhang et al. 2021). Fungal invasion patterns in beetle-infested hosts may have been successional. *Ips typographus*, for example, attacked Norway spruce with the virulent *Ceratocystispolonica*, followed by other beetle-diffused *Graphium* and *Ophiostoma* fungi (Solheim 1992a, 1992b); *Tomicusminor* invaded *Pinussylvestris* with *Hormonemadematioides* first, followed by *Ophiostomatingens* and *O.canum* (Jankowiak 2008). *Ophiostomaips* was not isolated from the 2^nd^–3^rd^ instar larvae galleries of *M.alternatus*, but the isolation rate from the 4^th^–5^th^ instar larvae galleries was 92.5% (Lun et al. 2019). The diversity and abundance of fungi associated with *M.alternatus* larvae of different instars in PWN-infected pines, as well as the successional pattern of fungi in PWN-*M.alteratus* symbionts are unknown. To date, 14 ophiostomatalean fungi have been obtained from *M.alternatus* galleries and pupal chambers along with PWN (Zhao et al. 2013, 2014, 2018; Wang et al. 2018; Lun et al. 2019). However, these studies are sporadic reports and no systematic studies have been conducted.

This research aimed to compare the diversity of fungi in different instars of the PWN-infected *M.alternatus* larval galleries and pupal chambers in south-eastern China. Field surveys were used in conjunction with integrated morphological observation and multi-locus DNA sequence analysis to describe the diversity of fungi associated with PWN and *M.alternatus*. This study provides a scientifically reliable and theoretical foundation for effective PWD control from the fungal perspective.

## ﻿Materials and methods

### ﻿Collection of samples and fungal isolations

From October to November 2020, fungi were isolated from 380 and 510 samples of different instars from *M.alternatus* larvae galleries and pupal chambers in *Pinusmassoniana*, respectively, in the Huangyan District (28°56'90"N, 121°17'56"E), Xianju County (28°75'28"N, 120°59'97"E), Zhejiang Province. All the trees used in this study showed signs of death and sap stains and PWNs were simultaneously isolated from galleries and pupal chambers by Behrman funnel. The samples were collected by hand saw, individually placed in sterile envelopes, stored at 4 °C and separated within a week. The surfaces of galleries and pupal chambers were disinfected with 1.5% sodium hypochlorite for 1 min, rinsed with sterile water three times and then cut into approximately 3 × 3 mm^2^ tissue blocks. They were then inoculated on to a 2% (w/v) water agar medium (20 g agar powder in 1 l of deionised water) and cultured in the dark at 25 °C (Seifert et al. 1993; Wang et al. 2019; Wang et al. 2020). Subsequently, all strains were purified by hyphal tip isolation (Eckhardt 2002) and transferred on to 2% (w/v) malt extract agar (MEA: 20 g malt extract powder and 20 g agar powder in 1 l of deionised water) for growth in the dark at 25 °C. All strains were deposited at the Chinese Academy of Forestry (Table 1). Representative cultures were deposited at the China Forestry Culture Collection Center (CFCC) (Table 2).

**Table 1.** Species of the fungi isolated from *Pinusmassoniana* infected by *Monochamusalternates* and *Bursaphelenchusxylophilus* in the current study.

Taxon	Species	2^nd^–3^rd^ instar larvae		4^th^~5^th^ instar larvae	
		number	isolation rate	number	isolation rate
1	*Ceratocystiopsisweihaiensis*	3	1.18%	N/A	N/A
	*Chaetomiumglobosum*	1	0.39%	N/A	N/A
	*Colletotrichumgloeosporioides*	8	3.14%	N/A	N/A
	*Cytospora* sp.	11	4.31%	N/A	N/A
	*Diplodiasapinea*	27	10.59%	76	16.74%
	*Fusarium* sp.	10	3.92%	N/A	N/A
2	*Graphilbum* sp.	N/A	N/A	12	2.64%
3	*Ophiostomaips*	N/A	N/A	231	50.88%
4	*Ophiostoma* sp.	62	24.31%	N/A	N/A
	*Penicillium* sp.	2	0.78%	5	1.10%
	*Pestalotiopsis* sp.	N/A	N/A	2	0.44%
	*Phialocephala* sp.	45	17.65%	N/A	N/A
	*Pseudocosmospora* sp.	14	5.49%	N/A	N/A
	*Schizophyllum* sp.	8	3.14%	N/A	N/A
5	*Sporothrixmacroconidia*	4	1.57%	N/A	N/A
6	*S.zhejiangensis*	8	3.14%	N/A	N/A
	*Trichodermaatroviride*	N/A	N/A	107	23.57%
	*Xenoacremonium* sp.	37	14.51%	N/A	N/A
	Unidentified	15	5.88%	21	4.63%
	The total number of strains	255	100%	454	100%

Unseparated data is represented by [N/A].

### ﻿Culture and morphological studies

The growth of representative strains was monitored daily and the culture characteristics of the colonies were recorded. Microscopic features were observed using a BX51 Olympus microscope (Tokyo, Japan) with differential interference contrast. Fifty measurements were made for each microscopic taxonomical structure. The formula (min–) (mean–SD)–(mean+SD) (–max) was used to calculate averages, ranges, standard deviation (SD), minimum (min) and maximum (max) measurements, respectively. All relevant data pertaining to type specimens were deposited in MycoBank (www. MycoBank.org).

**Table 2.** Strains of ophiostomatalean fungi isolated from *Pinusmassoniana* infested by *Monochamusalternatus* and *Bursaphelenchusxylophilus* in the current study.

Taxon	Species	Strain no	Location	GenBank no		
				ITS	TUB2	TEF1-α
1	*Ceratocystiopsisweihaiensis*	CFCC 55742 CXY4012	Huangyan	OK104016	OM103280	N/A
		CFCC 55743 CXY4013	Huangyan	OK104017	OM103281	N/A
		CXY4019	Huangyan	N/A	N/A	N/A
2	***Graphilbumxianjuensis* sp. nov.**	**CFCC 55738^T^ CXY4010**	Xianju	OK104014	OM103285	ON033177
		CFCC 55739 CXY4011	Xianju	OK104015	OM103286	ON033178
		CXY4018	Xianju	N/A	N/A	N/A
3	*Ophiostomaips*	CFCC 55735 CXY4005	Xianju	OK104009	OM056673	N/A
		CFCC 55736 CXY4006	Xianju	OK104010	OM056674	N/A
		CFCC 55732 CXY4007	Xianju	OK104011	OM056675	N/A
4	***Ophiostomataizhouense* sp. nov.**	**CFCC 55740^T^ CXC4001**	Huangyan	OK104005	OM103276	N/A
		CFCC 55731 CXY4002	Huangyan	OK104006	OM103277	N/A
		CFCC 55733 CXY4003	Huangyan	OK104007	OM103278	N/A
		CFCC 55734 CXY4004	Huangyan	OK104008	OM103279	N/A
5	*Sporothrixmacroconidia*	CFCC 55741 CXY4009	Huangyan	OK104013	OL352730	N/A
		CXY4016	Huangyan	N/A	N/A	N/A
		CXY4017	Huangyan	N/A	N/A	N/A
6	*S.zhejiangensis*	CFCC 55737 CXY4008	Huangyan	OK104012	OM103282	N/A
		CXY4014	Huangyan	N/A	OM103283	N/A
		CXY4015	Huangyan	N/A	OM103284	N/A

Species names in bold are novel species described in this study. “T” indicates ex-type strains. CFCC: China Forestry Culture Collection Center, Beijing, China. CXY (Culture Xingyao): Culture collection of the Research Institute of Forest Ecology and Nature Conservation, Chinese Academy of Forestry. Sequences missing data are indicated by [N/A]

A 7 mm diameter mycelium plug was taken from a flourishing fungal colony using a sterile puncher and placed at the centre of 90 mm diameter 2% MEA plates, with one side of mycelium in contact with the media. Five replicate plates for each strain were incubated in a dark incubator at 5–35 °C with a temperature interval of 5 °C. The diameter of the colonies on each dish was measured every day by the orthogonal method until the fastest-growing mycelium reached the edge of the dish. The colony colour was then described using the Rayner (1970) colour chart.

### ﻿DNA extraction, amplification and sequencing

Mycelia of representative strains were scraped with a sterile blade from the edge of the medium and transferred to 2 ml Eppendorf tubes for DNA extraction. DNA extraction and purification were carried out using the Invisorb Spin Plant Mini Kit (Tiangen, Beijing, China) according to the manufacturer’s instructions. The primer pairs ITS1/ITS4 (White et al. 1990), BT2a/BT2b (Glass and Donaldson 1995) and EF1/EF2 (Jacobs et al. 2004) were used for the internal transcribed spacer (ITS) region of the nuclear ribosomal DNA operon, including spacers 1 and 2 and the 5.8S gene, the β-tubulin (BT) gene region and transcription elongation factor-1α (TEF-1α), respectively.

Polymerase chain reaction (PCR) amplification was performed using a Veriti 96-Well Fast Thermal Cycler (Applied Biosystems Veriti96, Foster City, CA, USA). PCR was carried out in a final volume of 25 μl (2.5 mM MgCl_2_, 1× PCR buffer, 0.2 mM dNTP, 0.2 mM of each primer and 2.5 U Taq-polymerase enzyme). The cycling conditions were the same as those described for primer design (White et al. 1990; Glass and Donaldson 1995; Jacobs et al. 2004). The PCR products were purified using the MSB Spin PCR Apace Kit (250) (Invitek, Berlin, Germany) in accordance with the manufacturer’s instructions.

Sequencing reactions were performed using a CEQ DTCS Quick Start Kit (Beckman Coulter, Brea, CA, USA) according to the manufacturer’s instructions, with the same PCR primers as above. Nucleotide sequences were determined using a CEQ 2000 XL capillary automated sequencer (Beckman Coulter). Complementary and overlapping DNA electropherograms were checked and assembled using BioEdit v. 7.2.0. (Hall 1999).

### ﻿Sequence alignment and phylogenetic analysis

Preliminary identification of the strains was conducted using the standard basic local alignment search tool (BLAST) searches in NCBI GenBank (http://blast.ncbi.nlm.nih.gov/Blast.cgi) and sequences with the highest similarity were downloaded from GenBank. Alignment of the genes was performed using MAFFT 7.0 (https://mafft.cbrc.jp/alignment/server/) (Katoh and Standley 2013), with the E-INS-I option with a gap-opening penalty of 1.53 and an offset value of 0.00 and edited manually using Molecular Evolutionary Genetic Analyses (MEGA) 7.0 software (Kumar et al. 2016). Maximum Parsimony (MP), Maximum Likelihood (ML) and Bayesian Inference (BI) were used to infer the phylogenetic trees from each dataset. Two concatenated matrices of ITS and BT sequences were generated for *Ceratocystiopsis* and the *O.ips* complex.

MP analyses were implemented using PAUP* version 4.0b10 (Swofford 2003). The gaps were treated as the fifth base. Bootstrap analysis (1000 bootstrap repetitions) was used to determine the confidence level for inferring the nodes in the tree topology. Tree bisection and reconnection were selected as the branch swapping option. For each dataset of the 5000 most-parsimonious trees, the best tree that was automatically output by PAUP* v. 4.0b10 was selected for use in the figure.

ML analyses were carried out using RAxML-HPC (version 8.2.3; Stamatakis 2014) and the selected GTR-GAMMA model of site substitution included the estimation of gamma-distributed rate heterogeneity and a proportion of invariant sites. ML analysis included 1000 bootstrap analyses to evaluate the overall reliability of the node support value and tree topology.

BI analyses using Markov Chain Monte Carlo (MCMC) methods were implemented in MrBayes version 3.1.2 (Ronquist et al. 2012), from a random starting tree for 5,000,000 generations, to calculate posterior probability values for the nodes. When we run to 5,000,000 generations, the split frequencies of all datasets were less than 0.01. Chain convergence for all datasets was determined using Tracer 1.7 (Rambaut et al. 2018). No lack of convergence was detected. Trees were sampled every 100 generations and the first 25% of trees sampled were discarded as burn-in, while the remaining trees were used to calculate Bayesian posterior probabilities of the clades. Phylogenetic trees were edited in Figtree version 1.4.3 (http://tree.bio.ed.ac.uk/sosftware/figtree/) and Adobe Illustrator CS6.

## ﻿Results

### ﻿Fungal isolation and sequence comparison

A total of 709 strains of fungi were isolated from the *M.alternatus* larval galleries and pupal chambers (2^nd^–5^th^ instars). The strains were divided into 18 taxa, based on colony morphology and multi-locus DNA sequence alignment (ITS and BT) analysis. A total of 255 fungal strains, representing 14 taxa, were isolated from the galleries of 2^nd^–3^rd^ instar larvae. Taxon 4 was the dominant taxon accounting for 62 of the 255 strains. A total of 454 fungal strains were isolated from the galleries and pupal chambers of the 4^th^–5^th^ instar larvae and divided into six taxa. The dominant taxon was *O.ips*, accounting for 231 out of the 454 strains (Table 1). Only two fungi (*Diplodiasapinea* and *Penicillium* sp.) could be isolated from both 2^nd^–3^rd^ instar larval galleries and 4^th^–5^th^ instar larval galleries and pupal chambers, except for unidentified species. In this study, 320 ophiostomatalean fungi strains (320 strains out of 709 fungal strains) were isolated, including six tentative species. Four of these six species were obtained from the galleries of the 2^nd^–3^rd^ instar larvae and two species were isolated from the galleries and pupal chambers of the 4^th^–5^th^ instar larvae (Table 1).

### ﻿Phylogenetic analyses

There were 709 strains obtained in this study, but some strains have a small number of strains. In this study, we selected 2–4 representative strains from each Taxon and nineteen representative strains of Ophiostomatales belonging to six tentative species (Taxa 1–6) were selected for phylogenetic analyses (Table 2). All the sequences used for the phylogenetic trees were submitted to GenBank. The three phylogenetic approaches yielded similar topologies, with statistical support varying slightly for each sequence dataset. Phylograms derived from ML analysis were presented for each individual dataset, along with nodal supports derived from the MP and BI analyses.

**Figure 1.**
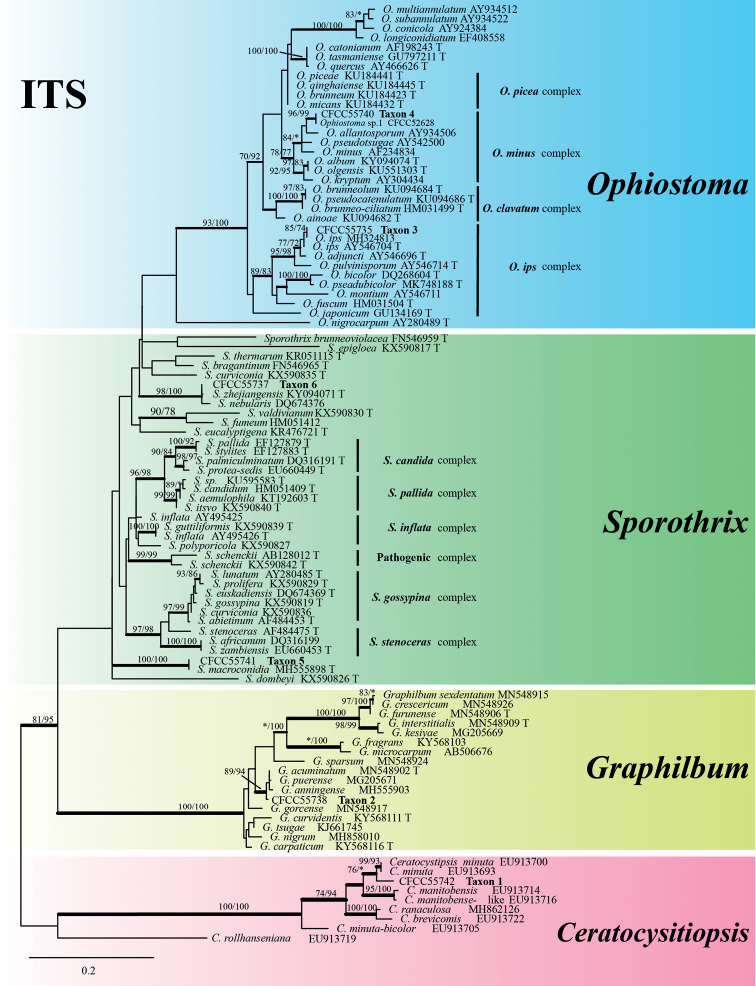
ML tree of the ITS region of *Ophiostoma*, *Sporothrix*, *Graphilbum*, *Ceratocystiopsis*. Bootstrap values of ML/MP ≥ 70% are recorded at nodes as ML/MP and bold branches indicate posterior probability values ≥ 0.9. ML and MP, Bootstrap values < 70% are indicated by the symbol *. The tree is drawn to scale (see bar) with branch length measured in the number of substitutions per site. Strains representing ex-type sequences are marked with “T.” ML, Maximum Likelihood; MP, Maximum Parsimony; BI, Bayesian Inference and the final alignment of 734 positions, including gaps.

The ITS phylogenetic tree showed that six representative taxa (Table 2) belonged to six phylogenetic clades (Fig. 1). Taxa 1–2 nested within the *Ceratocystiopsis* and *Graphilbum* lineages, respectively; Taxa 3–4 nested within the *Ophiostoma* lineage, Taxon 3 belonging to the *Ophiostomaminus* complex and Taxon 4 belonging to the *O.ips* complex (de Beer and Wingfield 2013); Taxa 5–6 belonged to the *Sporothrix* and were not placed in any complex defined by de Beer et al. (2016) (Fig. 1).

**Figure 2.**
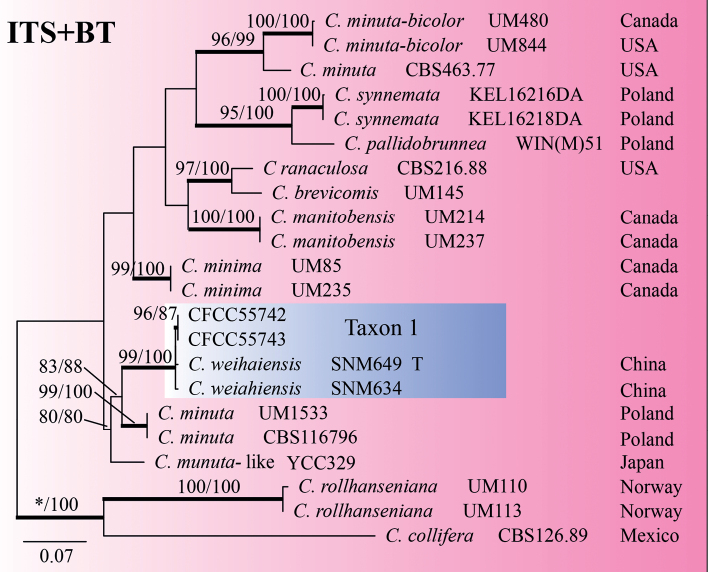
ML tree of *Ceratocystiopsis* generated from the combined (ITS+BT) sequence data. Bootstrap values of ML/MP ≥ 70% are recorded at nodes as ML/MP and bold branches indicate posterior probability values ≥ 0.9. ML and MP, Bootstrap values < 70% are indicated by the symbol *. The tree is drawn to scale (see bar) with branch length measured in the number of substitutions per site. Strains representing ex-type sequences are marked with “T.” Abbreviations: ML, Maximum Likelihood; MP, Maximum Parsimony; BI, Bayesian Inference and the final alignment of 1040 positions, including gaps.

Taxon 1 included three isolates, all of which were included in the analyses (Tables 1, 2). Based on the phylogenetic analysis of the combined dataset (ITS+BT), this taxon forms a well-supported clade with *Ceratocystiopsisweihaiensis* (Fig. 2). Hence, the strains in Taxon 1 should be identified as *C.weihaiensis*.

**Figure 3.**
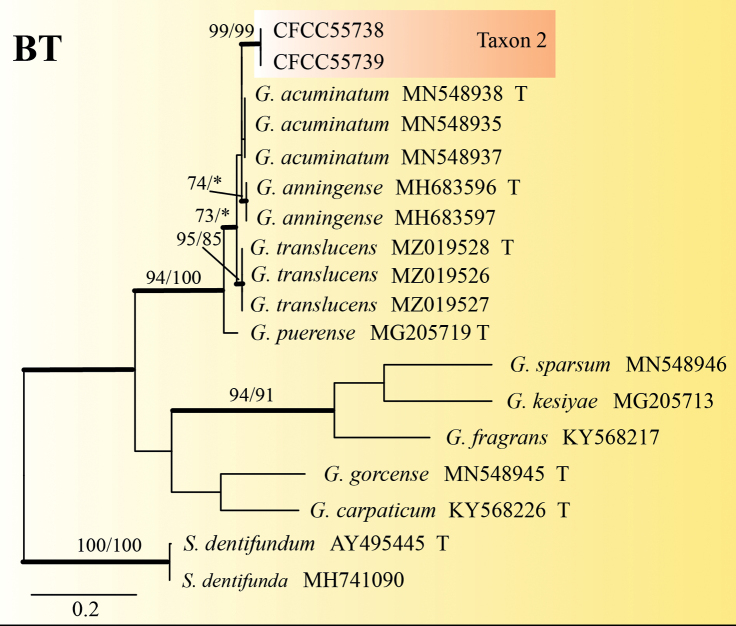
ML tree of the BT region of *Graphilbum*. Bootstrap values of ML/MP ≥ 70% are recorded at nodes as ML/MP and bold branches indicate posterior probability values ≥ 0.9. ML and MP, Bootstrap values < 70% are indicated by the symbol *. The tree is drawn to scale (see bar) with branch length measured in the number of substitutions per site. Strains representing ex-type sequences are marked with “T.” Abbreviations: ML, Maximum Likelihood; MP, Maximum Parsimony; BI, Bayesian Inference and the final alignment of 548 positions, including gaps.

**Figure 4.**
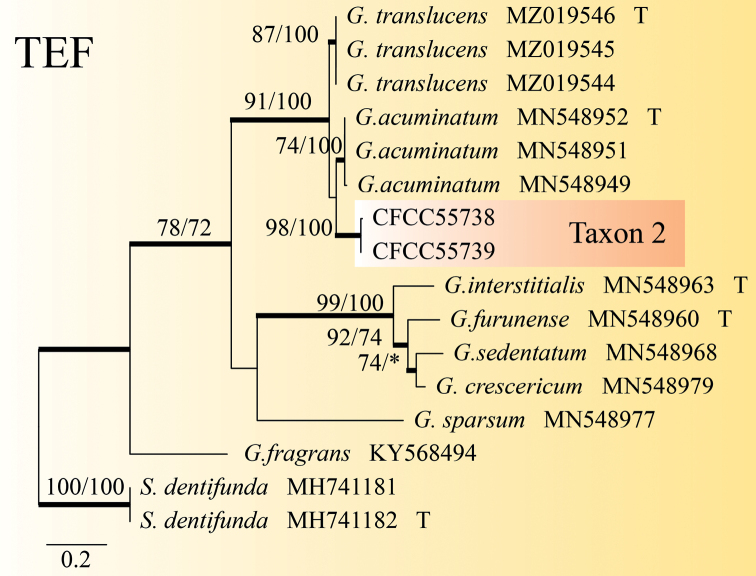
ML tree of the TEF region of *Graphilbum*. Bootstrap values of ML/MP ≥ 70% are recorded at nodes as ML/MP and bold branches indicate posterior probability values ≥ 0.9. ML and MP, Bootstrap values < 70% are indicated by the symbol *. The tree is drawn to scale (see bar) with branch length measured in the number of substitutions per site. Strains representing ex-type sequences are marked with “T.” Abbreviations: ML, Maximum Likelihood; MP, Maximum Parsimony; BI, Bayesian Inference and the final alignment of 725 positions, including gaps.

Taxon 2 consisted of 12 isolates, three of which were used for phylogenetic analyses (Tables 1, 2). The phylograms of ITS, BT and TEF-1α datasets revealed that Taxon 2 was an independent clade closely related to *Graphilbumacuminatum*, *G.anningense* and *G.translucens* (Figs 1, 3, 4). As a result, Taxon 2 should be interpreted as belonging to a distinct, undescribed *Graphilbum* species.

**Figure 5.**
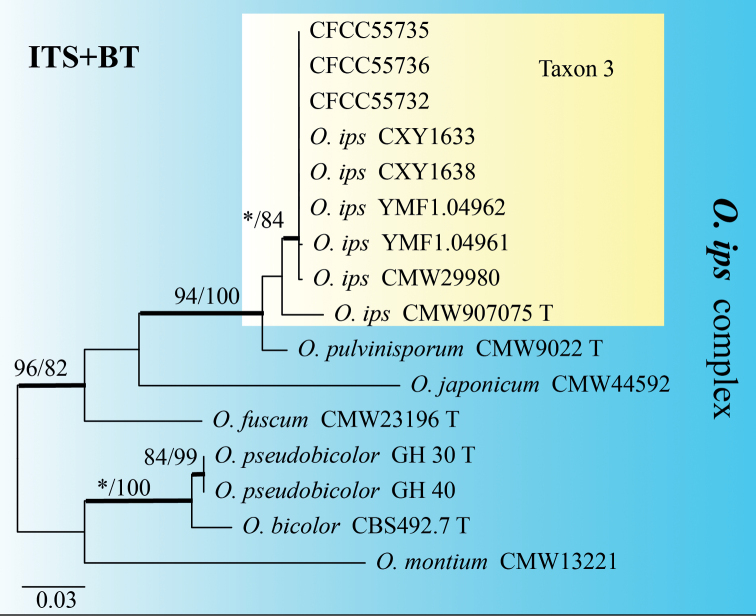
ML tree of the *O.ips* complex generated from the combined (ITS+BT) sequence data. Bootstrap values of ML/MP ≥ 70% are recorded at nodes as ML/MP and bold branches indicate posterior probability values ≥ 0.9. ML and MP, Bootstrap values < 70% are indicated by the symbol *. The tree is drawn to scale (see bar) with branch length measured in the number of substitutions per site. Strains representing ex-type sequences are marked with “T.” Abbreviations: ML, Maximum Likelihood; MP, Maximum Parsimony; BI, Bayesian Inference and the final alignment of 953 positions, including gaps.

**Figure 6.**
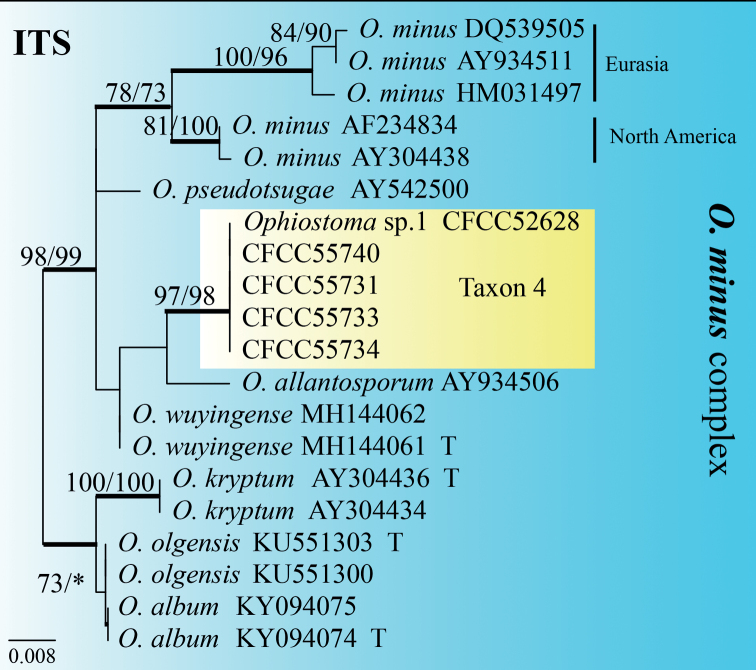
ML tree of the ITS region of *O.minus* complex. Bootstrap values of ML/MP ≥ 70% are recorded at nodes as ML/MP and bold branches indicate posterior probability values ≥ 0.9. ML and MP, Bootstrap values < 70% are indicated by the symbol *. The tree is drawn to scale (see bar) with branch length measured in the number of substitutions per site. Strains representing ex-type sequences are marked with “T.” Abbreviations: ML, Maximum Likelihood; MP, Maximum Parsimony; BI, Bayesian Inference and the final alignment of 537 positions, including gaps.

**Figure 7.**
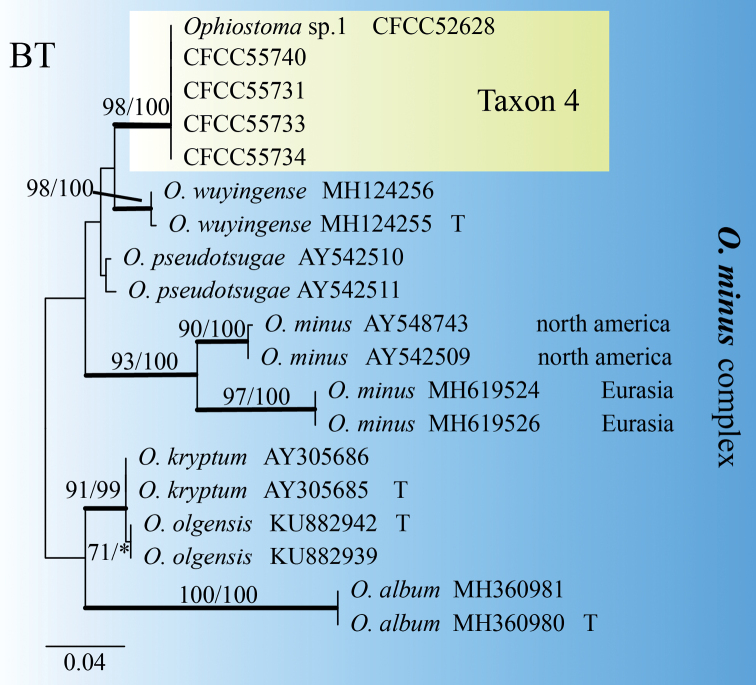
ML tree of the BT region of *O.minus* complex. Bootstrap values of ML/MP ≥ 70% are recorded at nodes as ML/MP and bold branches indicate posterior probability values ≥ 0.9. ML and MP, Bootstrap values < 70% are indicated by the symbol *. The tree is drawn to scale (see bar) with branch length measured in the number of substitutions per site. Strains representing ex-type sequences are marked with “T.” Abbreviations: ML, Maximum Likelihood; MP, Maximum Parsimony; BI, Bayesian Inference and the final alignment of 495 positions, including gaps.

Taxon 3 was represented by three sequences that formed a well-supported clade with *O.ips*, based on the ITS tree (Fig. 1). Further phylogenetic analysis, based on combined datasets (ITS+BT) yielded similar results (Fig. 5). Based on ITS and BT phylogenetic analysis, Taxon 4, represented by three sequences, has a well-supported independent clade with *Ophiostoma* sp.1 (CFCC52628) (Wang et al. 2019), which is closely related to *O.allantosporum*, *O.pseudotsugae* and *O.wuyingensis* (Figs 6, 7). Thus, Taxon 3 should be identified as a known species of *O.ips*, whereas Taxon 4 should be interpreted as a distinct, undescribed *Ophiostoma* species.

**Figure 8.**
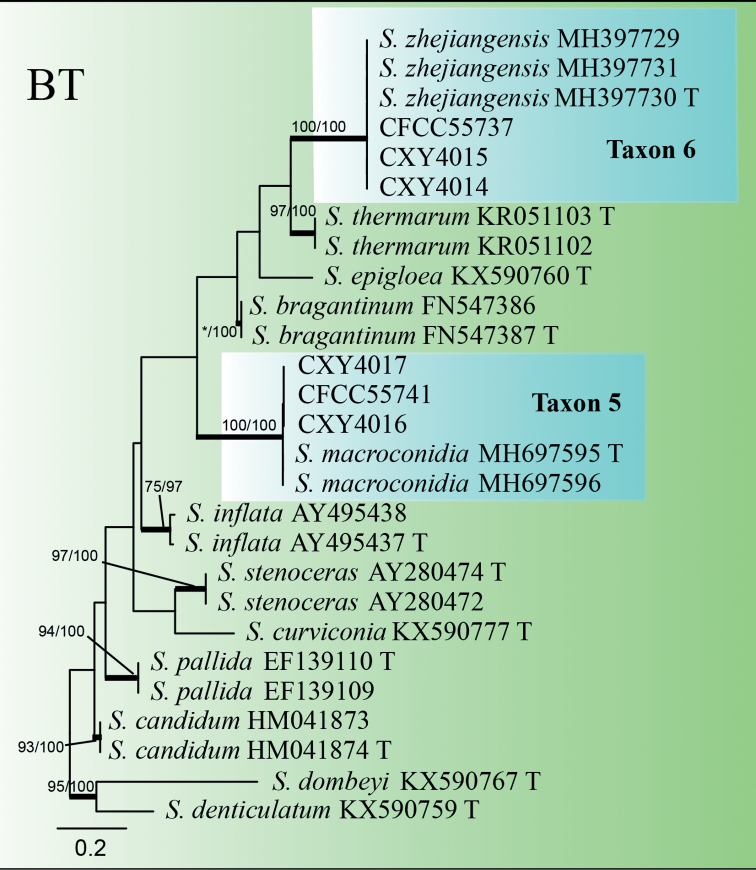
ML tree of *Sporothrix* generated from the BT sequence data. Bootstrap values of ML/MP ≥ 70% are recorded at nodes as ML/MP and bold branches indicate posterior probability values ≥ 0.9. ML and MP, Bootstrap values < 70% are indicated by the symbol *. The tree is drawn to scale (see bar) with branch length measured in the number of substitutions per site. Strains representing ex-type sequences are marked with “T.” Abbreviations: ML, Maximum Likelihood; MP, Maximum Parsimony; BI, Bayesian Inference and the final alignment of 313 positions, including gaps.

Taxon 5 consisted of four isolates, three of which were used for the phylogenetic analyses. Based on the ITS and BT phylogenetic trees, Taxon 5 grouped with *Sporothrixzhejiangensis* (Figs 1, 8). Thus, it should be identified as *S.zhejiangensis*.

Taxon 6 consisted of eight isolates, three of which were selected for analysis. Taxon 6 grouped with *Sporothrixmacroconidia*, based on the ITS and BT phylogenetic trees (Figs 1, 8). As a result, Taxon 6 was designated as *S.macroconidia*.

### ﻿Taxonomy

According to the ITS and BT phylogenetic analyses, six different taxa (Taxon 1–6) were identified in this study. They represent four known species, *Ceratocystiopsisweihaiensis*, *O.ips*, *S.macroconidia* and *S.zhejiangensis* (Lun et al. 2019; Wang et al. 2019; Chang et al. 2021), in addition to two novel species. They are described as follows:

#### ﻿ Graphilbum xianjuensis

Taxon classificationFungiOphiostomatalesOphiostomataceae

G. H. Zheng & Q. Lu sp. nov.

4242DC34-8587-5E3B-82DD-1778107FF107

 842387

Fig. 9

##### Etymology.

The epithet *xianju* (Latin) refers to the type locality.

##### Type.

China, Zhejiang, Xianju County, from *Monochamusalternatus* galleries and pupal chambers of *Pinusmassoniana* infested by *Bursaphelenchusxylophilus*, December 2020, collected by G. H. Zheng, culture ex-holotype CFCC55738 = CXY4010.

**Figure 9.**
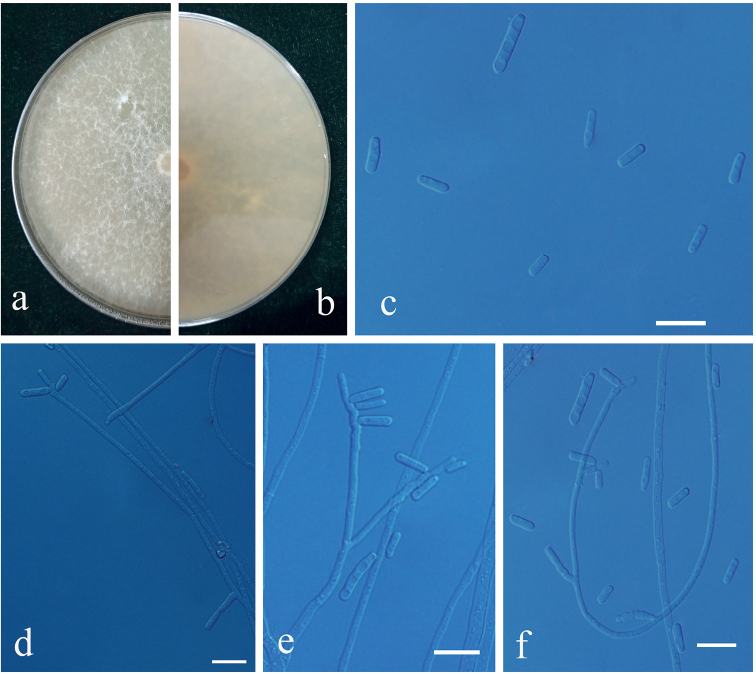
Morphological characteristics of *Graphilbunxianjuensis* sp. nov. (CFCC = 55738, Taxon 2). **a, b** thirty-day-old cultures on 2% MEA **c–f***Hyalorhincladiella*-like asexual morph: conidiogenous cells and conidia. Scale bars: 10 μm (**c–f**).

##### Description.

**Sexual morph**: not observed.

**Asexual form**: *Hyalorhincladiella*-like. Conidiogenous cells were simple or loosely branched, (9.12–) (15.44) – (48.64) (–62.49) × (1.25–) (1.53) – (2.21) (–2.45) μm. Conidia hyaline, smooth, cylindrical, aseptate, (4.76–) (6.07) – (9.87) (–13.41) × (0.99 –) (1.32) – (2.1) (–2.65) μm.

##### Culture characteristics.

Colonies on 2% MEA reaching 44.9 mm diameter, after incubation in the dark at 25 °C for 3 d, growth rate up to 14.98 mm/d at the fastest and colony margin irregular. Mycelium superficial to flocculose or floccose, hyaline, reverse grey-white. The optimal temperature for growth at 30 °C; no growth was observed at 5 °C.

##### Habitat and distribution.

Larval galleries and pupal chambers of *Monochamusalternatus* in *Pinusmassoniana*, infested by *Bursaphelenchusxylophilus*, in Zhejiang Province, China.

##### Additional specimens examined.

China, Zhejiang, from *Monochamusalternatus* galleries and pupal chambers of *Pinusmassoniana* infested by *Bursaphelenchusxylophilus*, December 2020, collected by G. H. Zheng, CFCC55739 = CXY4011, CXY4018.

##### Note.

Only the *Hyalorhincladiella*-like asexual form was observed in *Graphilbumxianjuensis*. This is closely related to *the G.acuminatum*, *G.anningense* and *G.translucens*, based on the ITS, BT and TEF1-α phylogenetic trees (Figs 1, 3, 4). Four species differed according to the size of their conidia. The conidia of *G.xianjuensis* (6.07–9.87 μm) are longer than those of *G.anningense* (4.5–6.4 μm), *G.acuminatum* (3.5–6 μm) and *G.translucens* (2.4–3.5 μm) (Wang et al. 2019; Jankowiak et al. 2020). Besides, *G.xianjuensis* was found to be associated with *M.alternatus* and PWN-infested *P.massoniana*, whereas *G.anningense* was reported in galleries of *T.yunnanensis* and *T.minor* associated with *P.yunnanensis* in southwest China (Wang et al. 2019), *G.acuminatum* has been reported in galleries of *Ips acuminatus* and *Pityogenesbidentatus* associated with *P.sylvestris* in Europe (Jankowiak et al. 2020) and *G.translucens* was first reported in *Cryphaluspiceae* associated with *P.densiflora*. In conclusion, four species of *Graphilbum* differ not only in geographical distribution, but also in hosts and vectors. The optimum growth temperature of *G.xianjuensis*, *G.anningense* and *G.translucens* is 30 °C and only *G.acuminatum* had an optimum growth temperature of 25 °C (Wang et al. 2019; Jankowiak et al. 2020).

#### ﻿ Ophiostoma taizhouense

Taxon classificationFungiOphiostomatalesOphiostomataceae

G. H. Zheng & Q. Lu sp. nov.

B2EC9688-0058-5EFC-99B6-CC662E651494

 842388

Fig. 10

##### Etymology.

‘*taizhou*’ (Latin) refers to the type locality.

##### Type.

China, Zhejiang Province, Taizhou City, from *Monochamusalternatus* galleries of *Pinusmassoniana* infested by *Bursaphelenchusxylophilus*, October 2020, collected by G. H. Zheng and Q. Lu, culture ex-holotype CFCC55740 = CXY4001.

**Figure 10.**
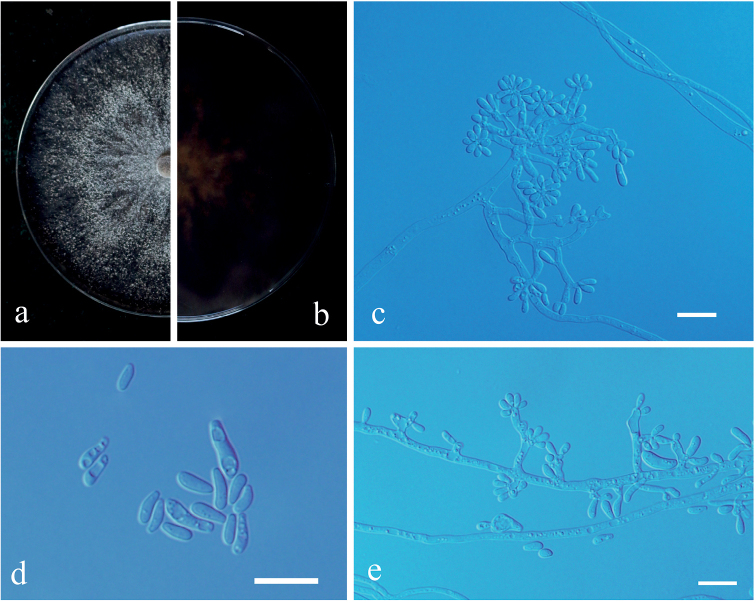
Morphological characteristics of *Ophiostomataizhouense* sp. nov. (CFCC = 55740, Taxon 4). **a, b** twenty-day-old cultures on 2% MEA **c–e***Hyalorhincladiella*-like asexual morph: conidiogenous cells and conidia. Scale bars: 10 μm (**c–e**).

##### Description.

**Sexual morph**: not observed.

**Asexual form**: *Hyalorhincladiella*-like. Conidiophores abundant, conidiogenous cells single, disposed in a dense rachis (3.08–) (6.6) – (15.63) (–23.07) × (1.11–) (1.44) – (2.23) (–2.9) μm. Conidia hyaline, smooth, lunate, ellipsoid to ovoid, curvulate, aseptate, (3.24–) (4.27) – (7.42) (–10.08) × (1.17–) (1.6) – (2.39) (–2.86) μm.

##### Culture characteristics.

Colonies on 2% MEA reaching 62.5 mm diameter, after incubation in the dark at 25 °C for 3 d, growth rate up to 22.83 mm/d at the fastest, colony margin smooth, hyphae are superficial on agar. Some white mycelium was produced early during growth and became black after 8–15 d, transitioning from brown to dark brown. The optimal temperature for growth at 30 °C; no growth was observed at 5 °C.

##### Habitat and distribution.

Larval galleries of *Monochamusalternatus* in *Pinusmassoniana*, infested by *Bursaphelenchusxylophilus*, in Zhejiang Province, China.

##### Additional specimen examined.

China, Zhejiang, Taizhou City, from *Monochamusalternatus* galleries of *Pinusmassoniana* infested by *Bursaphelenchusxylophilus*, October 2020, collected by G. H. Zheng and Q. Lu, CFCC55731 = CXY4002, CFCC55733 = CXY4003, CFCC55734 = CXY4004.

##### Note.

Only the *Hyalorhincladiella*-like asexual form was observed in *Ophiostomataizhouense*. According to ITS and BT phylogenetic analysis, it has a well-supported independent clade with *Ophiostoma* sp.1 (CFCC52628) and is closely related to *O.allantosporum*, *O.pseudotsugae* and *O.wuyingensis* (Figs 1, 5, 6). Only one strain of *Ophiostoma* sp.1 was isolated in our laboratory from *P.yunnanensis* infested with *T.yunnanensis* in Yunnan Province, so this strain was not officially named before this study. Although the geographical location and host of *O.taizhouense* and *Ophiostoma* sp.1 are different, their culture characteristics and gene sequences (ITS and BT) are identical (Figs 1, 5, 6) (Wang et al. 2019). In general, the conidia of *O.taizhouense* (4.27–7.42 μm) are longer than those of *O.minus* (2.5–6 μm) (Upadhyay 1981) and *O.pseudotsugae* (2.7–5 μm) (Rumbold 1936). The optimal growth temperature of *O.allantosporum* and *O.pseudotsugae* was 25 °C, that of *O.wuyingensis* was 25–30 °C and that of *O.taizhouense* was 30 °C (Gorton and Webber 2000, Chang et al. 2019). Both *O.wuyingensis* and *O.taizhouense* showed pigmentation on 2% MEA medium, whereas *O.allantosporum* has mid-brown hyphae, *O.pseudotsugae* has white-grey to snuff-brown, both showed no agar pigmentation (Rumbold 1936; Villarreal et al. 2005). *Ophiostomawuyingensis* was first isolated from the gallery of *Ips typographus* on *P.koraiensis* in Heilongjiang Province (Chang et al. 2019). *Ophiostomaallantosporum* and *O.pseudotsugae* were isolated from *P.resinosa* in the USA and *P.menziesii* were infected with *Dendroctonusfrontalis* in North America (Gorton and Webber 2000). Strains of *O.taizhouense* in this study were isolated from *P.massoniana* infected with PWN and *M.alternatus*.

## ﻿Discussion

**Figure 11.**
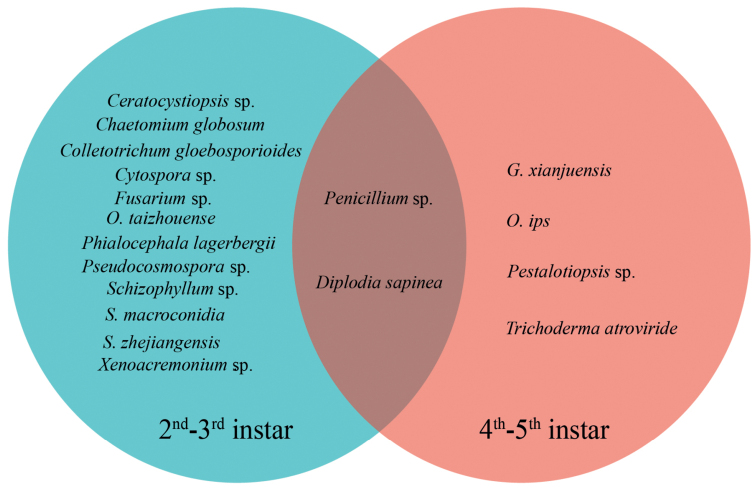
Diagram showing the species of fungi were isolated from the galleries and pupae chambers of different instar larvae of *Monochamusalternates*.

In the current study, 255 (containing 14 species) and 454 (containing six species) strains were obtained from *M.alternatus* larval galleries and pupal chambers of 2^nd^–3^rd^ and 4^th^–5^th^ instar, respectively, in *P.massoniana* infested with PWN in the Zhejiang Province, south-eastern China (Table 1). A total of 320 ophiostomatalean fungal strains out of overall 709 strains were obtained. The fungal diversity of 2^nd^–3^rd^ instar larvae was higher than that of 4^th^–5^th^ instar larvae (Table 1; Fig. 11). *Ophiostomataizhouense* is the dominant species in the 2^nd^–3^rd^ instar and *O.ips* is the primary species at the 4^th^–5^th^ instar. This is both similar and distinct from the previous research. Some studies found *Trichoderma* sp. or *Sporothrix* sp.1 to be the most common fungus associated with PWD (Zeng et al. 2006; Zhao et al. 2013), while others found the same to be *O.ips* (Lun et al. 2019), as was found here. The phenomenon could be caused by fungal succession, which occurs when PWN and *M.alternatus* select fungal companions that are more conducive to their own growth and reproduction at different life cycle stages. Therefore, future research on fungal diversity and abundance will necessitate a more comprehensive sampling analysis.

Only two common fungal species were obtained from both 2^nd^–3^rd^ instar larval galleries, 4^th^–5^th^ instar larval galleries and pupal chambers. The abundance of *D.sapinea* (103 out of 709) was higher than that of *Penicillium* sp. (7 out of 709). *Diplodiasapinea* is commonly isolated from *P.nigra* tip blight, *P.halepensis* and *P.pinaster* branch cankers worldwide (Luchi et al. 2014). It is an important pathogen of the *Pinus* spp. In addition, research has shown that *D.sapinea* can promote PWN reproduction and settlement (Kobayashi et al. 1974; Sriwati et al. 2007). *Penicillium* sp. is a common fungus in nature that also serves as an important biocontrol fungus (Sartaj et al. 2011; Win et al. 2021). However, there are no reports of *Penicillium* sp. affecting PWN, either negatively or positively.

*Ophiostomaips* was first reported in pine trees associated with bark beetles in the south-eastern United States (Rumbold 1936) and it has since been confirmed, using microsatellite markers, to be distributed worldwide (Zhou et al. 2007). Unfortunately, the study did not include Chinese strains. However, *O.ips* is regarded as one of the most stable natural associates of PWN in the wild of China (Zhao et al. 2013; Lun et al. 2019). According to Lun et al. (2019), *O.ips* was the dominant strain associated with PWN and was frequently isolated at the late stage of PWD. In a study by Zhao et al. (2013), *O.ips* was one of the three dominant ophiostomatoid fungi associated with PWN, with an isolation rate of 36%. Although *O.ips* was not found in the galleries of *M.alternatus* 2^nd^–3^rd^ instar larvae in this study, it was the primary species at the 4^th^–5^th^ instar, with an isolation rate of 50.88% and fungal abundance was much higher than that of other fungi during this period. Experiments with nematode propagation revealed that *O.ips* could breed nematodes, but not as effectively as *Botrytiscinerea* (Pimentel et al. 2021). In addition, biochemical analysis results revealed that *O.ips* could produce a wide range of volatile chemical substances (Cale et al. 2019). The 4^th^–5^th^ instar larvae of *M.alternatus* are closely related to pinewood nematode dispersal stage J_IV_ (the fourth-stage dispersal juvenile). However, the mechanism underlying the interaction between *O.ips* and dispersal nematode juveniles is still lacking.

*Ophiostomataizhouense* was the second most frequently isolated species of ophiostomatalean fungi in our study (62 out of the 255 strains); nevertheless, it was only associated with 2^nd^–3^rd^ instar larvae. The association between *O.taizhouense* and PWD needs further experimental verification as a new species. Although the isolation rate of *Phialocephala* sp. and *Xenoacremonium* sp. is relatively high in 2^nd^–3^rd^ instar larvae, these two fungi are both endophytes and there are no reports relating them to PWD.

In addition, three known ophiostomatalean fungi (*C.weihaiensis*, *S.macroconidia* and *S.zhejiangensis*) and one new species (*G.xianjuensis*) were revealed with low isolation rates during the survey. Simultaneously, some common endophytic and saprophytic fungi were isolated from the galleries and pupal chambers of *M.alternatus* larvae. The relationship between these fungi and PWN has not yet been reported.

In this study, a relatively large diversity of fungal species was obtained and identified as associated with PWN and *M.alternatus* in south-eastern China. The results showed that the fungal diversity and abundance of the 2^nd^–3^rd^ instar larvae differed from those of the 4^th^–5^th^ instar larvae. Fungi play an important role during the successful survival, reproduction and spread of PWN (Zhao et al. 2014; Zhao and Sun 2017). Hence, it is vital to explore the relationship between fungi and PWDs. This study provides a research basis for the fungi-PWN-*M.alternatus* symbiosis.

## Supplementary Material

XML Treatment for Graphilbum xianjuensis

XML Treatment for Ophiostoma taizhouense
